# Supplementary material for: Hybrid Models and Biological Model Reduction with PyDSTool
Source: PLoS Comput Biol. 2012 Aug 9;8(8):e1002628. doi: 10.1371/journal.pcbi.1002628 (PMC3415397; doi:10.1371/journal.pcbi.1002628)
Supplement: Text S4 — Complete source code for the PyDSTool package (version 0.88.120504). Includes API documentation and help files linking to web pages. This file is identical to the current public release on Sourceforge.net. (ZIP) [file pcbi.1002628.s004.zip › PyDSTool/html/PyDSTool.utils-module.html]

xml version="1.0" encoding="ascii"?


PyDSTool.utils


| Home | Trees | Indices | Help | PyDSTool |
| --- | --- | --- | --- | --- |

Package PyDSTool ::
Module utils

[hide private]

[frames] | no frames]

# Module utils

source code

User utilities.


|  |  |
| --- | --- |
| Functions | [hide private] |

|  |  |
| --- | --- |
| makeMfileFunction(name, argname, defs)  defs is a dictionary of left-hand side -> right-hand side definitions | source code |

|  |  |
| --- | --- |
| info(x, specName=`'``Contents``'`, offset=1, recurseDepth=1, recurseDepthLimit=2, \_repeatFirstTime=False)  Pretty printer for showing argument lists and dictionary specifications. | source code |

|  |  |
| --- | --- |
| makeImplicitFunc(f, x0, fprime=None, extrafargs=`(``)`, xtolval=1e-08, maxnumiter=100, solmethod=`'``newton``'`, standalone=True)  Builds an implicit function representation of an N-dimensional curve specified by (N-1) equations. | source code |

|  |  |
| --- | --- |
| findClosestPointIndex(pt, target, tol=inf, in\_order=True)  Find index of the closest N-dimensional Point in the target N by M array or Pointset. | source code |

|  |  |
| --- | --- |
| findClosestArray(input\_array, target\_array, tol)  Find the set of elements in (1D) input\_array that are closest to elements in target\_array. | source code |

|  |  |
| --- | --- |
| find(x, v, next\_largest=1, indices=None)  Returns the index into the 1D array x corresponding to the element of x that is either equal to v or the nearest to v. | source code |

|  |  |
| --- | --- |
| orderEventData(edict, evnames=None, nonames=False, bytime=False)  Time-order event data dictionary items. | source code |

|  |  |
| --- | --- |
| make\_RHS\_wrap(gen, xdict\_base, x0\_names, use\_gen\_params=False, overflow\_penalty=10000.0)  Return function wrapping Generator argument gen's RHS function, but restricting input and output dimensions to those specified by x0\_names. | source code |

|  |  |
| --- | --- |
| make\_Jac\_wrap(gen, xdict\_base, x0\_names, use\_gen\_params=False, overflow\_penalty=10000.0)  Return function wrapping Generator argument gen's Jacobian function, but restricting input and output dimensions to those specified by x0\_names. | source code |

|  |  |
| --- | --- |
| saveObjects(objlist, filename, force=False)  Store PyDSTool objects to file. | source code |

|  |  |
| --- | --- |
| loadObjects(filename, namelist=None)  Retrieve PyDSTool objects from file. | source code |

|  |  |
| --- | --- |
| intersect(a, b)  Find intersection of two lists, sequences, etc. | source code |

|  |  |
| --- | --- |
| union(a, b)  Find union of two lists, sequences, etc. | source code |

|  |  |
| --- | --- |
| remain(a, b)  Find remainder of two lists, sequences, etc., after intersection. | source code |

|  |  |
| --- | --- |
| compareList(a, b)  Compare elements of lists, ignoring order (like sets). | source code |

|  |  |
| --- | --- |
| cartesianProduct(a, b)  Returns the cartesian product of the sequences. | source code |

|  |  |
| --- | --- |
| makeDataDict(fieldnames, fieldvalues)  Zip arrays of field names and values into a dictionary. | source code |


|  |  |
| --- | --- |
| Variables | [hide private] |

all

predicate\_op

putmask

shape

complexfloating

DomainType

float32

float96

complex\_

ones

integer

API = `API_class()`

targetLangs = `['c', 'python', 'matlab']`

API\_class

argmax

sign

Macheps

Continuous = `Continuous Domain`

null\_predicate = `null_predicate_class(None)`

complex64

atleast\_1d

Discrete = `Discrete Domain`

float\_

less\_equal

float

log

complex128

int\_

complex192

int0

int8

double

finfo

spy

clip

int

logical\_or

deepcopy

floating

any

null\_predicate\_class

sqrt

complex

LargestInt32 = `2147483647`

fit\_function

int16

mat

npy

types

int64

exp

Inf = `inf`

NaN = `nan`

isfinite = `<ufunc 'isfinite'>`

less = `<ufunc 'less'>`

greater = `<ufunc 'greater'>`

\_classes = `[]`

\_functions = `['intersect', 'remain', 'union', 'cartesianProduc...`

\_mappings = `['_implicitSolveMethods', '_1DimplicitSolveMethods']`

rout = `redirc.Redirector(redirc.STDOUT)`

rerr = `redirc.Redirector(redirc.STDERR)`

\_implicitSolveMethods = `['newton', 'bisect', 'steffe', 'fsolve']`

\_1DimplicitSolveMethods = `['newton', 'bisect', 'steffe']`

\_all\_complex = `(<type 'complex'>, <type 'numpy.complexfloating...`

\_all\_float = `(<type 'float'>, <type 'numpy.floating'>, <type '...`

\_all\_int = `(<type 'int'>, <type 'numpy.integer'>, <type 'numpy...`

\_all\_numpy\_complex = `(<type 'numpy.complex128'>, <type 'numpy....`

\_all\_numpy\_float = `(<type 'numpy.float64'>, <type 'numpy.float...`

\_all\_numpy\_int = `(<type 'numpy.int32'>, <type 'numpy.int32'>, ...`

\_complex\_types = `(<type 'complex'>, <type 'numpy.complexfloati...`

\_float\_types = `(<type 'float'>, <type 'numpy.floating'>)`

\_int\_types = `(<type 'int'>, <type 'numpy.integer'>)`

\_num\_equivtype = `{<type 'float'>: <type 'numpy.float64'>, <typ...`

\_num\_maxmin = `{<type 'numpy.int32'>: [-2147483648, 2147483647]...`

\_num\_name2equivtypes = `{'float': (<type 'float'>, <type 'numpy...`

\_num\_name2type = `{'float': <type 'numpy.float64'>, 'int': <typ...`

\_num\_type2name = `{<type 'float'>: 'float', <type 'int'>: 'int'...`

\_num\_types = `(<type 'float'>, <type 'int'>, <type 'numpy.float...`

\_pytypefromtype = `{<type 'numpy.int32'>: <type 'int'>, <type '...`

\_real\_types = `(<type 'int'>, <type 'numpy.integer'>, <type 'fl...`

\_seq\_types = `(<type 'list'>, <type 'tuple'>, <type 'numpy.ndar...`


|  |  |
| --- | --- |
| Function Details | [hide private] |

|  |  |
| --- | --- |
| makeImplicitFunc(f, x0, fprime=None, extrafargs=`(``)`, xtolval=1e-08, maxnumiter=100, solmethod=`'``newton``'`, standalone=True) | source code |

Builds an implicit function representation of an N-dimensional curve
specified by (N-1) equations. Thus argument f is a function of 1
variable. In the case of the 'fsolve' method, f may have dimension up to
N-1.

Available solution methods are: newton, bisect, steffensen, fsolve.
All methods utilize SciPy's Minpack wrappers to Fortran codes.

Steffenson uses Aitken's Delta-squared convergence acceleration.
fsolve uses Minpack's hybrd and hybrj algorithms.

Standalone option (True by default) returns regular function. If
False, an additional argument is added, so as to be compatible as a
method definition.

|  |  |
| --- | --- |
| findClosestPointIndex(pt, target, tol=inf, in\_order=True) | source code |

Find index of the closest N-dimensional Point in the target N by M
array or Pointset. Uses norm of order given by the Point or Pointset,
unless they are inconsistent, in which case an exception is raised, or
unless they are both arrays, in which case 2-norm is assumed.

With the in\_order boolean option (default True), the function will
attempt to determine the local "direction" of the values and
return an insertion index that will preserve this ordering. This option
is incompatible with the tol option (see below).

If the optional tolerance, tol, is given, then an index is returned
only if the closest distance is within the tolerance. Otherwise, a
ValueError is raised. This option is incompatible with the in\_order
option.

|  |  |
| --- | --- |
| findClosestArray(input\_array, target\_array, tol) | source code |

```
Find the set of elements in (1D) input_array that are closest to
elements in target_array.  Record the indices of the elements in
target_array that are within tolerance, tol, of their closest
match. Also record the indices of the elements in target_array
that are outside tolerance, tol, of their match.

For example, given an array of observations with irregular
observation times along with an array of times of interest, this
routine can be used to find those observations that are closest to
the times of interest that are within a given time tolerance.

NOTE: input_array must be sorted! The array, target_array, does not have to be sorted.

Inputs:
  input_array:  a sorted float64 array
  target_array: a float64 array
  tol:          a tolerance

Returns:
  closest_indices:  the array of indices of elements in input_array that are closest to elements in target_array

Author: Gerry Wiener, 2004
Version 1.0
```

|  |  |
| --- | --- |
| find(x, v, next\_largest=1, indices=None) | source code |

Returns the index into the 1D array x corresponding to the element of
x that is either equal to v or the nearest to v. x is assumed to contain
unique elements.

if v is outside the range of values in x then the index of the
smallest or largest element of x is returned.

If next\_largest == 1 then the nearest element taken is the next
largest, otherwise if next\_largest == 0 then the next smallest is
taken.

The optional argument indices speeds up multiple calls to this
function if you have pre-calculated indices=argsort(x).

|  |  |
| --- | --- |
| orderEventData(edict, evnames=None, nonames=False, bytime=False) | source code |

```
Time-order event data dictionary items.

Returns time-ordered list of (eventname, time) tuples.

If 'evnames' argument included, this restricts output to only the named
  events.
The 'nonames' flag (default False) forces routine to return only the event
  times, with no associated event names.
The 'bytime' flag (default False) only works with nonames=False and returns
  the list in (time, eventname) order.
```

|  |  |
| --- | --- |
| make\_RHS\_wrap(gen, xdict\_base, x0\_names, use\_gen\_params=False, overflow\_penalty=10000.0) | source code |

```
Return function wrapping Generator argument gen's RHS function,
but restricting input and output dimensions to those specified by
x0_names. All other variable values will be given by those in xdict_base.
In case of overflow or ValueError during a call to the wrapped function,
an overflow penalty will be used for the returned values (default 1e4).

if use_gen_params flag is set (default False)
then:
  Return function has signature Rhs_wrap(x,t)
  and takes an array or list of x state variable values and scalar t,
  returning an array type of length len(x). The Generator's current param
  values (at call time) will be used.
else:
  Return function has signature Rhs_wrap(x,t,pdict)
  and takes an array or list of x state variable values, scalar t, and a
  dictionary of parameters for the Generator, returning an array type of
  length len(x).

NB: xdict_base will be copied as it will be updated in the wrapped
function.
```

|  |  |
| --- | --- |
| make\_Jac\_wrap(gen, xdict\_base, x0\_names, use\_gen\_params=False, overflow\_penalty=10000.0) | source code |

```
Return function wrapping Generator argument gen's Jacobian function,
but restricting input and output dimensions to those specified by
x0_names. All other variable values will be given by those in xdict_base.
In case of overflow or ValueError during a call to the wrapped function,
an overflow penalty will be used for the returned values (default 1e4).

if use_gen_params flag is set (default False)
then:
  Return function Jac_wrap(x,t) takes an array or list of x variable
  values and scalar t, returning a 2D array type of size len(x) by len(x).
  The Generator's current param values (at call time) will be used.
else:
  Return function Jac_wrap(x,t,pdict) takes an array or list of x variable
  values, scalar t, and a dictionary of parameters for the Generator,
  returning a 2D array type of size len(x) by len(x).

NB: xdict_base will be copied as it will be updated in the wrapped
function.
```

|  |  |
| --- | --- |
| saveObjects(objlist, filename, force=False) | source code |

Store PyDSTool objects to file. Argument should be a tuple or list,
but if a singleton non-sequence object X is given then it will be saved
as a list [ X ].

Some PyDSTool objects will not save using this function, and will
complain about attributes that do not have definitions in \_\_main\_\_.

|  |  |
| --- | --- |
| loadObjects(filename, namelist=None) | source code |

Retrieve PyDSTool objects from file. Returns list of objects unless
namelist option is given as a singleton string name. Also, if only one
object X was stored, it will be returned as [X], and thus you will have
to index the returned list with 0 to get X itself.

Optional namelist argument selects objects to return by name, provided
that the objects have name fields (otherwise they are ignored). If
namelist is a single string name then a single object is returned.

|  |  |
| --- | --- |
| intersect(a, b) | source code |

Find intersection of two lists, sequences, etc. Returns a list that
includes repetitions if they occur in the inputs.

|  |  |
| --- | --- |
| union(a, b) | source code |

Find union of two lists, sequences, etc. Returns a list that includes
repetitions if they occur in the input lists.

|  |  |
| --- | --- |
| remain(a, b) | source code |

Find remainder of two lists, sequences, etc., after intersection.
Returns a list that includes repetitions if they occur in the inputs.

|  |  |
| --- | --- |
| makeDataDict(fieldnames, fieldvalues) | source code |

Zip arrays of field names and values into a dictionary. For instance,
to use in Generator initialization arguments.

Deprecated as of v0.89.

  


|  |  |
| --- | --- |
| Variables Details | [hide private] |

### \_functions

Value:
:   ```
    ['intersect',
     'remain',
     'union',
     'cartesianProduct',
     'makeDataDict',
     'makeImplicitFunc',
     'orderEventData',
     'saveObjects',
    ...
    ```

### \_all\_complex

Value:
:   ```
    (<type 'complex'>,
     <type 'numpy.complexfloating'>,
     <type 'numpy.complex128'>,
     <type 'numpy.complex64'>,
     <type 'numpy.complex128'>)
    ```

### \_all\_float

Value:
:   ```
    (<type 'float'>,
     <type 'numpy.floating'>,
     <type 'numpy.float64'>,
     <type 'numpy.float32'>,
     <type 'numpy.float64'>)
    ```

### \_all\_int

Value:
:   ```
    (<type 'int'>,
     <type 'numpy.integer'>,
     <type 'numpy.int32'>,
     <type 'numpy.int32'>,
     <type 'numpy.int8'>,
     <type 'numpy.int16'>,
     <type 'numpy.int32'>,
     <type 'numpy.int64'>)
    ```

### \_all\_numpy\_complex

Value:
:   ```
    (<type 'numpy.complex128'>,
     <type 'numpy.complex64'>,
     <type 'numpy.complex128'>)
    ```

### \_all\_numpy\_float

Value:
:   ```
    (<type 'numpy.float64'>,
     <type 'numpy.float32'>,
     <type 'numpy.float64'>)
    ```

### \_all\_numpy\_int

Value:
:   ```
    (<type 'numpy.int32'>,
     <type 'numpy.int32'>,
     <type 'numpy.int8'>,
     <type 'numpy.int16'>,
     <type 'numpy.int32'>,
     <type 'numpy.int64'>)
    ```

### \_complex\_types

Value:
:   ```
    (<type 'complex'>, <type 'numpy.complexfloating'>)
    ```

### \_num\_equivtype

Value:
:   ```
    {<type 'float'>: <type 'numpy.float64'>,
     <type 'int'>: <type 'numpy.int32'>,
     <type 'numpy.integer'>: <type 'numpy.int32'>,
     <type 'numpy.floating'>: <type 'numpy.float64'>,
     <type 'numpy.int8'>: <type 'numpy.int32'>,
     <type 'numpy.int16'>: <type 'numpy.int32'>,
     <type 'numpy.int32'>: <type 'numpy.int32'>,
     <type 'numpy.int32'>: <type 'numpy.int32'>,
    ...
    ```

### \_num\_maxmin

Value:
:   ```
    {<type 'numpy.int32'>: [-2147483648, 2147483647],
     <type 'numpy.float64'>: [-inf, inf]}
    ```

### \_num\_name2equivtypes

Value:
:   ```
    {'float': (<type 'float'>,
               <type 'numpy.floating'>,
               <type 'numpy.float64'>,
               <type 'numpy.float32'>,
               <type 'numpy.float64'>),
     'int': (<type 'int'>,
             <type 'numpy.integer'>,
             <type 'numpy.int32'>,
    ...
    ```

### \_num\_name2type

Value:
:   ```
    {'float': <type 'numpy.float64'>, 'int': <type 'numpy.int32'>}
    ```

### \_num\_type2name

Value:
:   ```
    {<type 'float'>: 'float',
     <type 'int'>: 'int',
     <type 'numpy.integer'>: 'int',
     <type 'numpy.floating'>: 'float',
     <type 'numpy.int8'>: 'int',
     <type 'numpy.int16'>: 'int',
     <type 'numpy.int32'>: 'int',
     <type 'numpy.int32'>: 'int',
    ...
    ```

### \_num\_types

Value:
:   ```
    (<type 'float'>,
     <type 'int'>,
     <type 'numpy.floating'>,
     <type 'numpy.integer'>)
    ```

### \_pytypefromtype

Value:
:   ```
    {<type 'numpy.int32'>: <type 'int'>,
     <type 'numpy.float64'>: <type 'float'>}
    ```

### \_real\_types

Value:
:   ```
    (<type 'int'>,
     <type 'numpy.integer'>,
     <type 'float'>,
     <type 'numpy.floating'>)
    ```

### \_seq\_types

Value:
:   ```
    (<type 'list'>, <type 'tuple'>, <type 'numpy.ndarray'>)
    ```

  


| Home | Trees | Indices | Help | PyDSTool |
| --- | --- | --- | --- | --- |

|  |  |
| --- | --- |
| Generated by Epydoc 3.0.1 on Fri May 4 15:24:06 2012 | http://epydoc.sourceforge.net |
